# Synbiotic (FamiLact) administration in idiopathic male infertility enhances sperm quality, DNA integrity, and chromatin status: A triple-blinded randomized clinical trial

**DOI:** 10.18502/ijrm.v19i3.8571

**Published:** 2021-03-21

**Authors:** Behzad Abbasi, Homayoun Abbasi, Hassan Niroumand

**Affiliations:** ^1^Trauma Research Center, AJA University of Medical Sciences, Tehran, Iran.; ^2^Isfahan Fertility and Infertility Center, Isfahan, Iran.

**Keywords:** Male infertility, DNA fragmentation, Sperm, Synbiotics, Probiotics, Fertility agents.

## Abstract

**Background:**

Idiopathic male infertility is often treated empirically. A recent body of evidence has indicated the association between pro ± prebiotics administration and improvement in semen parameters.

**Objective:**

To assess the effect of FamiLact (probiotic + prebiotic) administration on male subjects with idiopathic infertility.

**Materials and Methods:**

Fifty-six men with idiopathic male infertility were randomly/equally divided into two groups. Men in the case and control groups received 500 mg of FamiLact and an identical placebo for 80 days, respectively. A semen sample was obtained from each of the participants before initiation and after the termination of the treatment course. Samples underwent regular semen analysis and were further analyzed to assess the level of DNA damage (sperm chromatin structure assay), oxidative stress (BODIPY C11 staining), and protamine deficiency (chromomycin-A3 staining) in spermatozoa.

**Results:**

No significant difference was observed between the baseline values of both groups. After intervention, mean sperm concentration, motility, and normal morphology were significantly higher in the FamiLact group compared to the placebo group (p < 0.05). In the FamiLact receivers, we detected improvement regarding the following parameters: concentration, motility, abnormal morphology, sperm lipid peroxidation, and DNA fragmentation (p ≤ 0.02). Likewise, in the placebo group, we noticed a decrease in the post-medication mean value of DNA fragmentation (p = 0.03) while observing no significant difference regarding other parameters.

**Conclusion:**

FamiLact administration improves sperm concentration, motility, and abnormal morphology and decrease sperm DNA damage, possibly through alleviating oxidative stress in the seminal fluid.

## 1. Introduction

A male factor contributes to 40-50% of all infertility cases and is the sole cause behind 20-30% of them (1). Abnormality in semen parameters leading to eventual sperm dysfunction is almost unanimously inseparable from male infertility etiologies. Today, the existing diagnostic modalities fail to point out the exact origins of abnormal semen parameters in a significant proportion of cases, a condition addressed as idiopathic male infertility (2). Although studies have proposed numerous contributing factors, they often share a common denominator: seminal oxidative stress (OS) (3). In the seminal plasma, disharmony between oxidizing agents and reductant molecules (i.e., antioxidants)-a state in which the former outbalances the latter- precipitates OS (4). OS may take place whether as a consequence of redundancy in seminal reactive oxygen species (ROS), generated dominantly by either sperm with abnormal morphology or leukocytes, or due to shortage in endo-/exogenous antioxidants (4).

Idiopathic male infertility is often treated empirically, employing hormonal and or nonhormonal remedies. Nonhormonal therapies commonly rely on oral supplementation with antioxidant compounds, exerting beneficial effects on sperm quality, pregnancy rate, live birth rate, and spermic DNA damage [for a recent review, check (5)]. Recently, performing random semen analyses for three Flortec (probiotic + prebiotic; Bracco, Italy) consumers diagnosed with gastrointestinal dysbiosis, Maretti and colleagues detected an unforeseen improvement in their sperm quality (6), the observation which prompted a pilot controlled clinical trial to evaluate the efficacy of probiotic administration on idiopathic oligoasthenoteratozoospermia. Supplementation with Flortec led to a statistically significant increase in the mean ejaculate volume, sperm concentration, progressive motility, and the percentage of morphologically normal spermatozoa (6). In a simultaneous attempt, Valcarce and colleagues underlined a significant decrease in sperm DNA damage in the medication period and an elevation in the percentage of motile sperms following probiotic administration on asthenozoospermic men (7).

Probiotics are live microorganisms that if administered adequately promote the host's well-being through various mechanisms, namely reinforcing the epithelial barrier and modifying the immune system (8). However, the mechanisms by which probiotics favorably contribute to male fertility are still arguable.

Pro-/prebiotic administration for male fertility purposes is a relatively novel field of research, and the existing evidence is scarce. Opting FamiLact, we aimed to further investigate the efficacy of supplementation with synbiotic products in idiopathic male infertility and the underlying mechanism(s). FamiLact is a synbiotic product containing a broad spectrum of beneficial *Lactobacillus* strains, *Bifidobacterium breve/longum*, and *Streptococcus thermophiles* accompanied by fructooligosaccharides as the prebiotic. Applying chromomycin A3 (CMA3) staining technique, this is the first study to evaluate the effects of such products on spermic DNA integrity by means of nuclear protamine content. Moreover, recruiting a relatively larger sample size compared to the pre-existing trials, we targeted more pragmatic results regarding DNA fragmentation.

## 2. Materials and Methods

### Patient selection

Through this triple-blind, randomized, placebo-controlled clinical trial, we selected the patients from the infertile couples' male partners, referred to the Imam Reza Hospital (Tehran, Iran) between November 2019 and May 2020. We defined infertility as the failure to establish a clinical pregnancy following 12 months of regular, unprotected sexual intercourse (9). According to the World Health Organization's designated cut-offs (10), men with sperm concentration < 15 (10${}^{6}$/mL) and/or normal morphology < 4 (%) and/or total motility < 40 (%) were considered eligible to participate in the study (solely addressed as oligozoospermia, teratozoospermia, and asthenozoospermia, respectively). As idiopathic male infertility is a diagnosis of exclusion, men with the following conditions were excluded from the study: cryptorchidism; varicocele; chromosome abnormalities; leukocytospermia; epididymal-orchitis; genito-urinary traumas; prostatitis; testicular torsion; history of inguinal/genital surgery; history of hormone therapy; endocrinopathies; history or ongoing use of cytotoxic drugs as well as immunosuppressants, anticonvulsants, and androgens; and recent history of sexually transmitted infections.

### Study design

**Figure 1:**
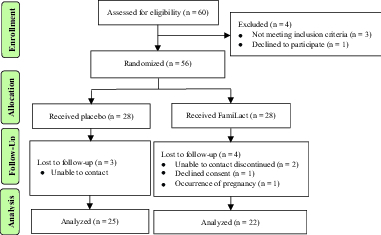
Flow diagram of the study according to CONSORT 2010.

Out of the 60 eligible patients, 56 met our inclusion criteria and were randomly divided into treatment (n = 28) and control (n = 28) groups. While the subjects in the treatment group were designated to receive a single capsule (500 mg) of FamiLact on a daily basis for 80 consecutive days, the controls took the identical placebo (Figure 1) (11). FamiLact is formulated by Zist Takhmir Pharmaceutical Company, under the permission of the Food and Drug Department of Iran's Ministry of Health and Medical Education (reference no.: 0347756442342525). Each capsule of FamiLact contains bacterial strains of *Lactobacillus rhamnosus*, *Lactobacillus casei*, *Lactobacillus bulgaricus*, *Lactobacillus acidophilus*, *Bifidobacterium breve*, *Bifidobacterium longum*, *Streptococcus thermophilus* (10${}^{9}$ CFU), and fructooligosaccharides as prebiotic.

Randomization was carried out utilizing the permutation block method: a random sequence on all possible permutations was obtained (nine blocks containing eight units). Drug and placebo, equal in packing and weight, were given to the participants according to the randomization sequence.

Patients, healthcare providers, data collectors, and statistical analysts were blinded to the randomization sequence.

### Sample collection and conventional semen analysis

Two semen samples were retrieved from each of the subjects before and 80 days after the onset of medication, provided by masturbation following two-seven days of sexual abstinence. Samples were left to liquefy in the ambient temperature. A trained operator, blinded to the medication sequence, fixed and analyzed the samples according to the World Health Organization's instructions. Sperm concentration was evaluated by applying a Sperm Counting Chamber (Shivani Scientific Industries, Mumbai, India), and motility was assessed through a computer-assisted sperm analysis system (Video Test, Version Sperm 2.1©, Russia).

Diff-Quik, CMA3, and BODIPY staining techniques were exerted to measure the morphological alterations, level of protamination, and lipid peroxidation status of the samples, respectively. In addition, the DNA fragmentation was evaluated by sperm chromatin structure assay (SCSA).

### Evaluating DNA fragmentation: SCSA

After segregating 2 million sperms from the samples, a buffer containing TNE/NaCl/EDTA was added to the vessel to increase the volume to 1 mL. In the case tube, 400 μl acid-detergent solution was added to 200 μl of the attenuated semen sample and was later mixed with 1200 μl of acridine orange staining solution (Sigma, St. Louis, USA), however, in the control tube the first step was bypassed. Next, the level of DNA fragmentation (percentage) was evaluated using a FACSCalibur flow cytometer (Becton Dickinson, San Jose, CA, USA). An approximate number of 10,000 spermatozoa/sample were analyzed.

### Evaluating lipid peroxidation: BODIPY probing

Nearly 2 million spermatozoa were isolated. BODIPY 581/591 C11 probe (D3861, Molecular Probes) [5 mM/ml] was added to the sample. Acquired tubes were then incubated for 30 min (37°C) followed by phosphate-buffered saline wash out. The percentage of BODIPY-positive spermatozoa (i.e., BODIPY-stained spermatozoa) was reported applying a FACSCalibur flow cytometer (Becton Dickinson, San Jose, CA, USA) (12).

### Evaluating protamine deficiency: CMA3 staining

Obtained samples were washed applying phosphate-buffered saline, followed by fixation in Carnoy's solution (methanol: glacial acetic acid 3:1; Merck, Germany). After that, two smears were provided and stained with CMA3 solution for 20 min and washed subsequently. For each sample, a minimum of 300 sperm cells was counted using a fluorescence microscope (Olympus BX51, Tokyo, Japan) with 460-470 nm filters. Sperms with bright yellow heads and the ones lacking brightness (dim yellow) were considered as CMA3-positive and -negative, respectively, and their proportion was further reported as percentages.

### Ethical considerations

This study was approved by AJA University of Medical Sciences Ethics Committee (IR.AJAUMS.REC.1398.051). Our trial was registered in the Iranian Registry of Clinical Trials (IRCT20190824044599N1). In addition, the course of the study was explained to all participants, and a signed formal consent was obtained from each of them.

### Statistical analysis

Statistical analysis was performed utilizing STATA v.13 (STATA Corp., Texas, USA) for Mac. We evaluated the distributional status of the variables drawing standardized normal probability plots. As perceived normally distributed, the inter- and intra-group comparison was done using the classical *t* test (mean comparison test) and paired *t* test, respectively. The calculated p-values < 0.05 were considered significant. All results were rendered as mean ± standard deviation (SD).

## 3. Results

After allocation, seven participants failed to attend the follow-up sampling. Additionally, one patient discontinued due to the occurrence of pregnancy and one declined consent, confining our study population to 47 subjects consisting of 22 cases and 25 controls (Figure 1). The mean age in the FamiLact and placebo groups was 34.5 and 33.8, respectively (p = 0.61).

As shown in Table I, the participants' baseline sperm characteristics did not show any statistically significant difference comparing the two study groups. However, in the endpoint semen samples, we noticed a significant dominance in the case group compared with the control group with respect to the concentration (p = 0.01), motility (p = 0.04), and normal morphology (p = 0.03) (Table I). Paired *t* test analysis revealed a significant favorable difference between the mean pre-/post-medication values in concentration (p = 0.004), motility (p = 0.003), abnormal morphology (p = 0.014), sperm lipid peroxidation (p = 0.02), and DNA fragmentation (p = 0.005) in the FamiLact receivers (Table II).

**Figure 2:**
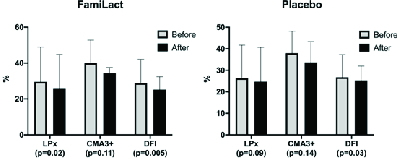
The effect of FamiLact supplementation vs placebo on the seminal OS, protamine deficiency, and DNA damage indicators in men with idiopathic infertility. Data are presented as Mean ± SD (paired *t* test). LPx: Lipid peroxidation, CMA3+: Chromomycin A3 positivity, DFI: DNA Fragmentation Index.

In contrast, the mean semen volume and CMA3 positivity did not differ significantly from the baseline measures. Likewise, we observed a decreased mean DNA fragmentation value in the placebo group after medication (p = 0.03), while other parameters showed no significant discrepancies (Figure 2).

**Table 1:** Baseline and endpoint sperm characteristics of the participants


	**FamiLact (n = 22)**		**Placebo (n = 25)**		
**Parameter**	**Before**	**After**	**Before**	**After**	**Intergroup p-value**
		2.75 ± 1.46	2.97 ± 2.22	2.54 ± 1.18		2.54 ± 1.86	0.59${}^{b}$
**Volume (ml)**	P = 0.47${}^{c}$		P = 0.97${}^{c}$		0.23${}^{a}$
		28.85 ± 17.1	44.1 ± 24.97	21.54 ± 15.48	27.98 ± 17.42	0.13${}^{b}$
**Concentration**	P = 0.004${}^{c}$		P = 0.06${}^{c}$		0.01${}^{a}$
		38.4 ± 25.08	50.81 ± 34.94	33.54 ± 20.3	34.44 ± 30.31	0.46${}^{b}$
**Motility (%)**	P = 0.003${}^{c}$		P = 0.39${}^{c}$		0.04${}^{a}$
		86.62 ± 11.61	79.5 ± 5.54	85.25 ± 9.61	83.5 ± 8.21	0.66${}^{b}$
**Abnormal morphology (%)**	P = 0.01${}^{c}$		P = 0.25${}^{c}$		0.03${}^{a}$
	Data are presented as Mean ± SD, ${}^{a}$Two sample *t* tests (comparing intergroup after-intervention values), ${}^{b}$Two sample *t* tests (comparing intergroup before-intervention values), ${}^{c}$Paired *t* test (comparing intragroup before/after values)					

**Table 2:** Level of lipid peroxidation, protamine deficiency, and DNA damage before and after the intervention


	**FamiLact (n = 22)**		**Placebo (n = 25)**		
**Parameter**	**Before**	**After**	**Before**	**After**	**Intergroup p-value**
		29.53 ± 19.4	26 ± 18.82	26.32 ± 15.35		24.72 ± 15.91	0.53${}^{b}$
**Sperm lipid peroxidation (%)**	P = 0.02${}^{c}$		P = 0.09${}^{c}$		0.8${}^{a}$
		39.97 ± 12.83	34.33 ± 3.24	37.97 ± 10.18	33.67 ± 9.69	0.27${}^{b}$
**CMA3 positivity (%)**	P = 0.11${}^{c}$		P = 0.14${}^{c}$		0.76${}^{a}$
		28.81 ± 13.27	25.19 ± 7.22	26.75 ± 10.54	25.32 ± 6.66	0.55${}^{b}$
**DNA fragmentation index (%) [SCSA]**	P = 0.005${}^{c}$		P = 0.03${}^{c}$		0.47${}^{a}$
	Data are presented as Mean ± SD, ${}^{a}$Two sample *t* tests (comparing intergroup after-intervention values), ${}^{b}$Two sample *t* tests (comparing intergroup before-intervention values), ${}^{c}$Paired *t* test (comparing intragroup before/after values), CMA3: Chromomycin A3, SCSA: Sperm chromatin structure assay					

## 4. Discussion

Through the course of this study, we aimed to explore the effect of FamiLact supplementation on sperm health. Our analysis indicated a significant improvement in sperm concentration, motility, abnormal morphology, and seminal lipid peroxidation, as well as sperm chromatin structure following 80 days of FamiLact administration (500 mg/daily) in male subjects with idiopathic infertility.

Intestinal microbiota, a vast number of specific microorganisms preoccupying the human's gastrointestinal tract, favorably contribute to the host's well-being through various mechanisms: reinforcing the epithelial barrier, competing with pathogens for adhesion to the receptors, regulating the immune system, and generating vitamins as well as short-chain fatty acids (13). When proportionally imbalanced (i.e., dysbiosis), gut microbiota fails to exert beneficial interactions with the host leading to alterations in the host's homeostasis (13). Probiotics are “live microorganisms that, when administered in adequate amounts, confer a health benefit on the host" (8). Inhabiting the gastrointestinal tract, probiotics outnumber the pathogens leading to the eventual amelioration in the epithelial barrier, immunomodulation, and production of the short-chain fatty acids and vitamins (13-15). Additionally, probiotics potentially target organs besides the gastrointestinal tract, mediated by immunoregulatory interactions and neurotransmitter production (16, 17).

FamiLact is a synbiotic product containing a broad spectrum of beneficial *Lactobacillus* strains, *Bifidobacterium breve/longum*, and *Streptococcus thermophiles* accompanied by fructooligosaccharides as prebiotics, “a substrate that is selectively utilized by host microorganisms conferring a health benefit” (18). The prebiotic content of the synbiotics, designated to promote the survival of probiotic strains in the gastrointestinal tract, synergically provides better functionality compared to the probiotic or prebiotic sole products (19).

To date, studies addressing the association between probiotic administration and sperm quality consist of few animal models and two exclusive studies recruiting humans as subjects. Although distinctive in design, these works share results in favor of probiotic supplementation regarding the enhancement in conventional semen parameters (6, 7, 20-24). Likewise, in our controlled clinical trial on men with idiopathically impaired sperm analysis, we noticed a significant increase in sperm concentration, motility, and normal morphology following an 80-day course of FamiLact supplementation. Consistent with our results, Valcarce and colleagues indicated an intense sixfold improvement in the sperm motility applying *Lactobacillus rhamnosus CECT8361* and *Bifidobacterium longum CECT7347* as the probiotic strains in 9 asthenozoospermic patients (7). Similarly, in a randomized clinical trial study consisting of 20 Flortec- and 21 placebo- (starch) receivers, Maretti and colleagues underlined a significant increase in the mean sperm progressive motility, as well as concentration, normal morphology, total count, and semen volume in the case subpopulation after six months of medication, while the before/after values did not differ significantly in the control group (6).

Besides, in light of the growing evidence, OS continues to strengthen its position as the dominant etiology behind idiopathic male infertility. Today, 30-80% of infertile men possess relatively increased seminal ROS levels (3, 25). Recently, Agarwal and colleagues have suggested a new terminology regarding the association between infertility and OS-male oxidative stress infertility (MOSI) - describing the coexistence of impairment in semen characteristics and OS in infertile men, many of which were diagnosed with idiopathic male infertility (3). Sperm DNA damage is among the several mechanisms by which OS may impair sperm's fertilizing capability (25, 26). Damaged DNA may further impair the implantation and development of the resultant embryo, and alter pregnancy and live birth rates (26). In the present study, we detected a significant decrease in DNA fragmentation index in both the FamiLact and placebo receivers (Figure 2). The observed alleviation in the DNA damage among the control group may be explained by plausible collateral supplementation, which may have been concealed from the research team; similarly, the analysis has revealed an almost-significant increase in the mean sperm concentration among the placebo receivers. However, the reduction in DNA damage was dominantly more significant in the FamiLact group (p = 0.005 vs. p = 0.03). Consistent with our result, the two mentioned trials observed similar results regarding DNA damage, albeit performing the analysis on confined numbers of the participants (Maretti and colleagues: n = 4; Valcarce and colleagues: n = 6-8) (6, 7). To acknowledge the interactions behind the association between synbiotic supplementation and DNA integrity, we monitored the alterations in the protamine content of the sperm chromatin. Protamine is a positively charged nuclear protein that replaces sperm nucleo-histones throughout a complicated process amid spermatogenesis leading to favorable condensation of the sperm genetic material (27). Condensed sperm chromatin is less vulnerable to damage, namely fragmentation (28). Utilizing the CMA3 staining technique, we observed a minimal non-significant decrease in protamine deficiency (i.e., CMA3 positivity) after the intervention (p = 0.11). This finding suggests that the resultant decline in DNA fragmentation following probiotic administration may not be mediated by increment in the protamine content, supporting the OS hypothesis as the underlying mechanism.

FamiLact contains different strains of lactobacilli (*L. rhamnosus*, *L. casei*, *L. bulgaricus*, and *L. acidophilus*) and bifidobacteria (*B. breve*, *B. longum)*. Evidence on OS-diminishing capabilities of probiotics is emerging (29, 30). However, such traits may be attributed to specific strains. Of note, lactobacilli and bifidobacteria enhance total antioxidant capacity and reduce markers of the systemic OS through several possible mechanisms, namely scavenging ROS or acting as a metal chelator (29). As demonstrated by a mice model study, probiotic supplementation may subside the free radical content of the seminal fluid, leading to the consequent alleviation in OS and eventual reduction in the spermic DNA damage (31). Consistent with this finding, we observed a significant decline in the percentage of sperm lipid peroxidation - as an indicator for the extent of OS-induced damage - in the FamiLact group after medication (p = 0.02).

## 5. Conclusion

FamiLact administration improves semen parameters, namely concentration, motility, and abnormal morphology, and reduces sperm DNA damage, possibly through alleviating OS state in the seminal fluid. Accordingly, synbiotic products -or more precisely, FamiLact- could be considered as a safe and affordable treatment for idiopathic male infertility. However, further investigation on pregnancy outcomes of synbiotic-treated patients may shed light on the fertility-related practical capabilities of such products.

##  Conflict of Interest

None.
